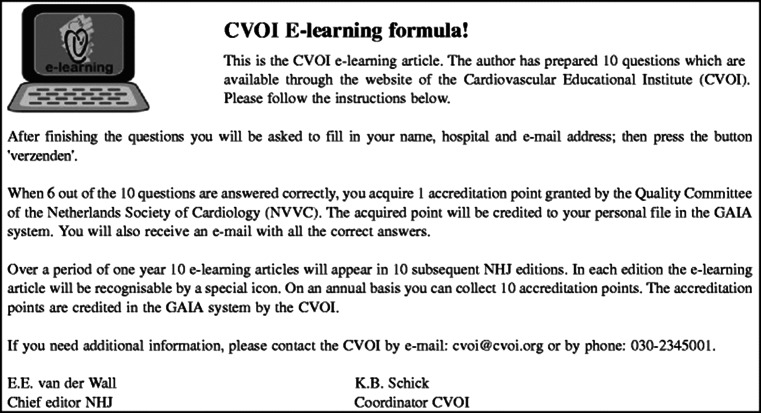# “First experience with JenaValve™: a single-centre cohort”

**DOI:** 10.1007/s12471-014-0619-8

**Published:** 2014-10-18

**Authors:** V. J. Nijenhuis, M. J. Swaans, V. Michiels, T. de Kroon, R. H. Heijmen, J. M. ten Berg

**Affiliations:** 1 Department of Cardiology, St. Antonius Hospital Koekoekslaan 1, 3435 Nieuwegein, the Netherlands; 2 Department of Cardiothoracic Surgery, St. Antonius Hospital, Nieuwegein, the Netherlands

**Keywords:** Aortic stenosis, Transcatheter aortic valve implantation, Transapical, TAVI, Jenavalve, PVL, Complication

## Abstract

**Aims:**

Since the introduction of transcatheter aortic valve implantation (TAVI), newer generation and novel devices such as the retrievable JenaValve™ have been developed. We evaluated the procedural and 6-month results of our first experience with implantation of the JenaValve™.

**Methods and results:**

From June 2012 to December 2013, 24 consecutive patients (mean age 80 ± 7 years, 42 % male) underwent an elective transapical TAVI with the JenaValve™. Device success was 88 %. The mortality rate was 4 % at 30 days and 31 % at 6 months. TAVI reduced the mean transvalvular gradient (44.2 ± 11.1 mmHg vs. 12.3 ± 4.3 mmHg, *p* < 0.001) and increased the mean aortic valve area (0.8 3 ± 0.23 to 1.70 ± 0.44 cm^2^). A mild paravalvular leakage (PVL) occurred in 4 patients (18 %) and a moderate PVL in 1 patient (4 %). Mean New York Heart Association Functional Class improved from 2.9 ± 0.5 to 2.0 ± 0.8 at 30 days.

**Conclusion:**

TAVI using the JenaValve™ prosthesis seems adequate and safe in this first experience cohort.

## Introduction

Currently, transcatheter aortic valve implantation (TAVI) is routine therapy in inoperable or high-risk patients with severe aortic stenosis [1, 2]. Two prostheses are predominantly used in clinical practice: the balloon-expandable SAPIEN XT™ bioprosthesis (Edwards Lifesciences, Irvine, USA), and the self-expandable CoreValve™ bioprosthesis (Medtronic, Minneapolis, USA). Newer developments focus on overcoming certain drawbacks of the SAPIEN XT™ and CoreValve™ prostheses, such as the limited capability of positioning and repositioning, retrieval, and the relatively high rate of periprosthetic paravalvular leakage (PVL). One such novel device is the JenaValve™ (JenaValve Technology GmbH, Munich), which received its CE approval for aortic stenosis in September 2011 and aortic regurgitation in September 2013. In this paper, we present the results of our first experience with implantation of the JenaValve™ for aortic stenosis.

## Methods

### Definitions

Endpoints were defined according to the updated Valve Academic Research Consortium (VARC)-2 [3] Consensus Document. The severity of PVL was determined by colour Doppler using transthoracic and transoesophageal echocardiography. The degree of PVL was classified as: 0 (none or trace), I (mild), II (moderate), or III (severe). Operative risk was determined with the Logistic EuroSCORE, EuroSCORE II, and the Society of Thoracic Surgeons (STS) score.

Bleeding complications were classified according to the consensus report from the Bleeding Academic Research Consortium (BARC) [4]. Acute kidney injury was classified using the Acute Kidney Injury Network (AKIN) classification system [5]. Early safety was defined as freedom of all-cause mortality, stroke, life-threatening bleeding, acute kidney injury (AKIN class II or III including renal replacement therapy), coronary artery obstruction requiring intervention, major vascular complication as defined by VARC-2 [3], and device-related valvular dysfunction requiring repeat procedure. Early safety was measured at 30 days. Procedure-related death was defined as all-cause mortality ≤30 days or during index hospitalisation if >30 days. Device success was defined as freedom of procedure mortality, correct positioning of the closure device, and the intended performance of the closure device.

### Patient population

**Fig. 1:**
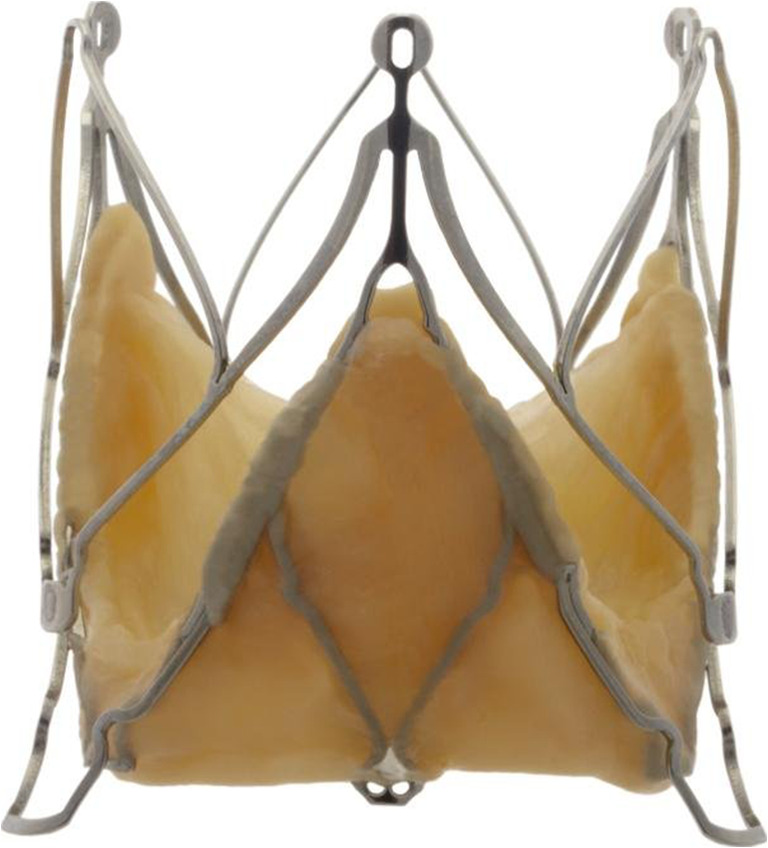
JenaValve™ prosthesis. Source: JenaValve Technology GmbH, Munich

From June 2012 to December 2013, we included all patients who underwent a TAVI using a JenaValve™ prosthesis (Fig. 1) in our institution. Patients were not eligible for transfemoral TAVI because of peripheral artery disease. All patients were discussed in a dedicated heart team consisting of at least one cardiothoracic surgeon, interventional cardiologist, and imaging specialist. Patients were considered ineligible for surgery because of a too high procedural risk, and were informed about the considerations and risks prior to intervention.

### Procedure

All procedures were performed under general anaesthesia in a specialised fully equipped hybrid catheterisation laboratory. Prophylactic antibiotics were given prior to the procedure. A temporary pacemaker lead was positioned transjugular in the right ventricle and tested at 180 bpm.

The common femoral artery was punctured using the standard technique. After administration of 5000 IU of unfractionated heparin with an activated clotting time ≥250 s, a pigtail catheter was advanced through a 5-French sheath into the ascending aorta for haemodynamic measurements and angiographic control. The optimal angulation of the fluoroscopic system was selected in concordance with pre-procedural computed tomographic (CT) imaging measurements. The fluoroscopic plane was perpendicular to the aortic annulus, whereby all three aortic valve leaflets were aligned and the course of the coronary arteries was identified.

The cardiac apex was exposed using a small left anterolateral thoracotomy between the fifth and sixth rib by a cardiothoracic surgeon, allowing direct visualisation for opening the pericardium. Purse string sutures with 2 × 4 pledgets were applied. The left ventricle was then punctured and a 14-French sheath was advanced into the left ventricular cavity using Seldinger’s technique. A J-curved stiff guide wire was advanced through the native aortic valve into the descending aorta. A straight valvuloplasty balloon was advanced over the wire and positioned within the stenotic aortic valve. Balloon diameter was chosen to be 2–5 mm smaller than the native aortic annulus measured by transoesophageal echocardiography. To predilate the stenotic aortic valve, standard apical aortic valvuloplasty was performed during rapid right ventricular pacing at 180–220 bpm.

**Fig. 2:**
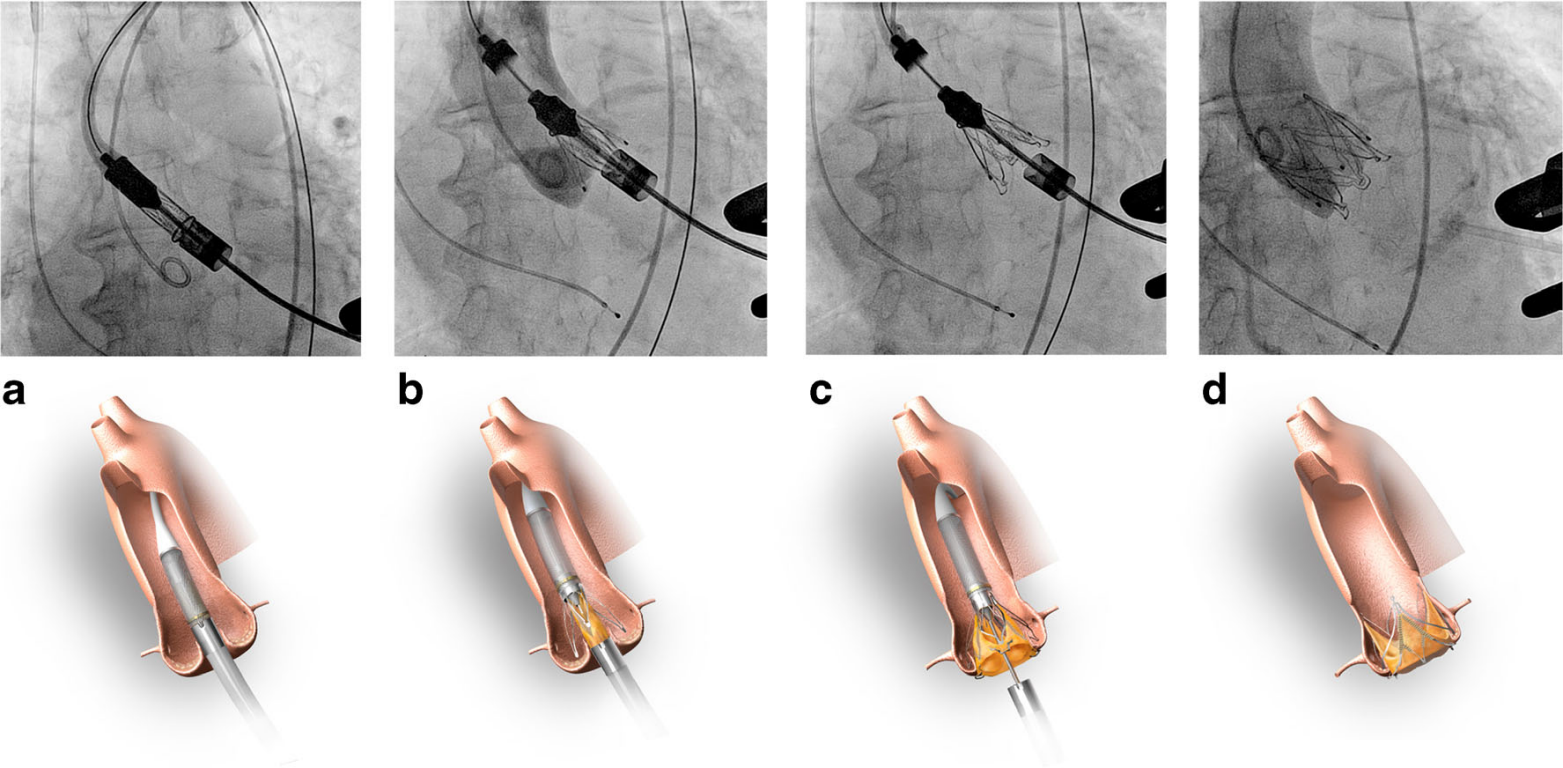
Stepwise implantation of the JenaValve™ prosthesis. Inferior images adapted with permission from JenaValve Technology GmbH, Munich

After pre-dilatation, the 14-French sheath was exchanged for the 32-French delivery sheath through which the JenaValve™ prosthesis was carefully advanced into the native aortic valve (Fig. 2a). The prosthesis was sized according to the annular perimeter on CT: a 23 mm valve for a perimeter of ≤72 mm, a 25 mm valve for a perimeter of 72 to 78.5 mm, and a 27 mm valve for a perimeter ≥78.5 mm. Once the distal catheter was in a supra-valvular position, we opened the sheath releasing the three ‘positioning feelers’. Subsequently, the catheter was carefully retracted, bringing each arm in the corresponding aortic sinus (Fig. 2b). This manoeuvre was performed under careful fluoroscopic guidance, verifying the correct position by the conjoint movement of each free arm in synchrony with each heartbeat. The device was released by careful unsheathing (Fig. 2c and d).

Fluoroscopic imaging and transoesophageal echocardiography were used to assess haemodynamic function and positioning of the valve in relation to the anatomic landmarks. An invasive transaortic pressure gradient was measured. In case of a high aortic peak gradient or a moderate to severe PVL, post-dilatation was performed. After retraction of the delivery system, haemostasis was secured by the purse-string sutures. When good haemostasis was achieved, a thoracic drain was placed. After standard wound closure, the patient was extubated and transferred to the post-anaesthetic care unit.

### Follow-up

Before discharge, a transthoracic echocardiography was routinely performed. Follow-up was performed at one and six months post-procedure.

### Data analysis

Data are presented as mean ± standard deviation or as median with interquartile range (IQR) as appropriate. Categorical variables are shown as frequencies and percentages. For pre-post comparison we used the paired sample *T*-test or the Wilcoxon signed-rank test as appropriate. Statistical significance was inferred at *p* < 0.05.

## Results

### Patient characteristics

Twenty-four patients (mean age 80 ± 7 years, 42 % male) underwent a transapical TAVI using the JenaValve™ prosthesis in our centre. All patients suffered from severe symptomatic aortic stenosis. NYHA functional class was ≥ II in all patients: class II in four patients (17 %), class III in 18 patients (75 %), and class IV in two patients (8 %). Impaired left ventricular ejection fraction (LVEF) was present in four patients (17 %). Mean logistic EuroSCORE was 25 ± 12 %, EuroSCORE II 5 ± 5 %, and STS score 5 ± 3 %. Demographic and baseline characteristics are listed in Table 1.

**Table 1:** Baseline characteristics (*n* = 24)

	All patients (n) %
Age (years)	80 ± 7
Female	(14) 58
BMI (kg/m^2^)	27 ± 4
Logistic EuroSCORE (%)	25 ± 12
EuroSCORE II (%)	5 ± 5
STS score (%)	5 ± 3
NYHA class	
II	(4) 17
III	(18) 75
IV	(2) 18
LVEF	
>50	(20) 83
31–50	(3) 13
<30	(1) 4
CAD	(15) 63
Myocardial infarction	(4) 17
CABG	(9) 38
Stroke	(5) 21
PAD	(10) 42
PHT	(6) 25
COPD	(5) 21
Diabetes mellitus	(7) 29
CKI	(2) 8
Atrial fibrillation	(7) 29
Permanent pacemaker	(2) 8
Medication	
Aspirin	(16) 67
Coumadin	(7) 29
Clopidogrel	(6) 25
Ticagrelor	(0) 0
Prasugrel	(2) 8
Dipyridamole	(3) 13

*BMI*, Body mass index; *CABG*, coronary artery bypass graft; *CAD*, coronary artery disease; *CKI*, chronic kidney injury; *COPD*, chronic obstructive pulmonary disease; *LVEF*, left ventricular ejection fraction; *NYHA*, New York Heart Association; *PAD*, peripheral artery disease; *PHT*, pulmonary hypertension; *STS*, Society of Thoracic Surgeons

### Procedure

A JenaValve™ prosthesis was successfully implanted in 21 patients (88 %). Of the unsuccessful procedures, one patient died due to an unknown cause at day 17, one patient had a moderate residual PVL, and one patient had an aortic mean gradient >20 mmHg. Procedural results are listed in Table 2.

**Table 2:** Procedural characteristics

	All patients (n) %
Implanted valve size	
23-mm	(6) 25
25-mm	(12) 50
27-mm	(6) 25
Post-dilatation	(16) 67
Conversion to open surgery	(0) 0
Valve-in-valve	(0) 0
Coronary ostium occlusion	(0) 0
Vascular complication	
False aneurysm	(1) 4
Composites	
Device success	(21) 88
Early safety	(21) 88

Categorical values are n (%)

Post-dilatation was used in 16 patients (67 %). We encountered no cases of valve malpositioning, unplanned cardiopulmonary bypass was not used, and there was no need for further interventions such as TAVI-in-TAVI or conversion to open surgery.

### Safety

Thirty-day mortality was 4 % (95 % CI 1 to 20 %) (*N* = 1). This patient died of an unknown cause. The composite safety endpoint was met in 21 patients (88 %, 95 % CI 70 to 96 %). Three patients did not meet this endpoint. Besides one death, an ischaemic stroke occurred in one patient (4 %) at day 0 complicated with a right-sided hemiparesis and dysarthria. This patient received aspirin and was loaded with clopidogrel (300 mg) the day before TAVI. Acute Kidney Injury Network (AKIN) class II occurred in 1 patient (4 %) (raise of creatinine from 98 to 278 μmol/L) due to forward failure and dehydration. Creatinine improved compared with before the procedure in 12 (50 %) patients.

Peri-procedure, a non-ST-elevation myocardial infarction occurred in 1 patient (4 %) at day 0. A post-procedural delirium occurred in three patients (13 %). During hospitalisation, a new left bundle branch block (LBBB) occurred in nine patients (38 %) and a pacemaker was implanted in three patients (13 %). Regarding bleeding, no life-threatening bleeding occurred. Major bleeding occurred in one patient (4 %) with diverticulitis who developed major gastrointestinal bleeding which required two blood transfusions. Minor bleeding occurred in three (13 %) patients and consisted of a femoral arterial bleed, gastrointestinal bleeding in a patient with ulcerative colitis, and a small transapical bleed observed in the thoracic drain. A major vascular access site-related complication occurred in one patient (4 %) who developed a false aneurysm of the femoral artery. Two patients (8 %) who had no complications were discharged without antiplatelet therapy. Complications are listed in Table 3.

**Table 3:** Echocardiographic parameters

	Baseline (*n* = 23)	Discharge (*n* = 22)	30 Days (*n* = 18)	*p*
LVEF	59 ± 15	59 ± 10	59 ± 14	0.78
Maximal aortic velocity	407 ± 70	259 ± 55	256 ± 45	<0.01
Maximal aortic PG	70 ± 22	28 ± 12	27 ± 10	<0.01
Mean aortic PG	42 ± 14	14 ± 6	14 ± 5	<0.01
AVA	0.83 ± 0.23	1.79 ± 0.59	1.70 ± 0.44	<0.01
Aortic regurgitation				0.13
Valvular AR (mild)	(5) 22	(0) 0	(0) 0	
Paravalvular AR (mild)	(0) 0	(4) 18	(3) 17	
Paravalvular AR (moderate)	(0) 0	(1) 4	(1) 6	
PASP	34 ± 16	37 ± 14	33 ± 11	0.50

Categorical values are n (%). *P* values signify paired test between baseline and discharge. *AR*, aortic regurgitation; *AVA*, aortic valve area; *LVEF*, left ventricular ejection fraction; *PG*, peak gradient; *PASP*, pulmonary artery systolic pressure

### Functional

**Fig. 3:**
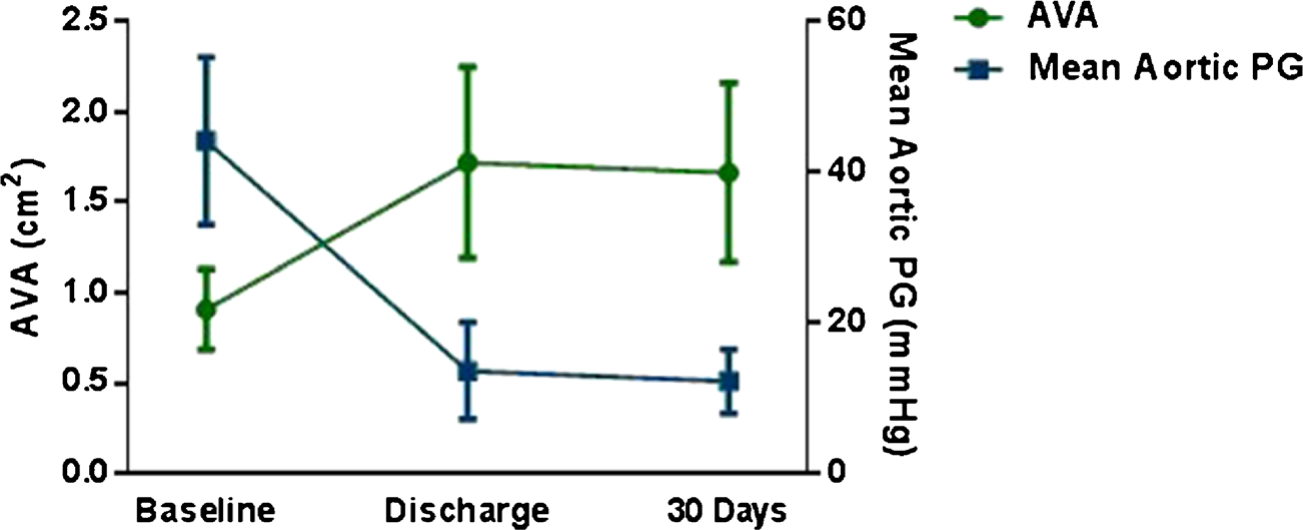
Echocardiographic assessment at baseline, discharge and 30 days of follow-up for mean aortic peak gradient (PG) and aortic valve area (AVA)

The mean NYHA functional class improved from 2.9 ± 0.5 to 2.0 ± 0.8 at 30 days (*P* < 0.001). Transvalvular pressure gradients significantly decreased by approximately 40 mmHg aortic peak gradient and 30 mmHg aortic mean gradient (*P* < 0.001). The mean aortic valve area increased from 0.83 ± 0.23 to 1.70 ± 0.44 cm^2^ (*P* < 0.000). A mild PVL occurred in 4 patients (18 %) and a moderate PVL in 1 patient (4 %). The mean LVEF remained stable. Echocardiographic parameters are displayed in Table 4 and Fig. 3.

**Table 4:** Major complications

	30 days (*n* = 24)	6 months (*n* = 16)
Death		
All-cause death	(1) 4	(5) 31
Cardiovascular death	(1) 4	(2) 13
Stroke	(1) 4	(1) 6
Myocardial infarction	(1) 4	(1) 6
Acute kidney injury		
AKIN I	(2) 8	–
AKIN II	(1) 4	–
Bleeding		
Major	(1) 4	(1) 6
Minor	(3) 13	(3) 19
New AF	(2) 8	–
New LBBB	(9) 38	–
New pacemaker	(3) 13	(4) 25

Major complications at mean follow-up (168 ± 30 days). Categorical values are n (%). *AF*, atrial fibrillation; *AKIN*, acute kidney injury network; *LBBB*, left bundle branch block

### Follow-up

Patients were discharged from hospital after a median of 7 days (IQR 5 days). Median stay in intensive care was 1 day (IQR 1 day). Median follow-up was four (IQR 12) months. Six-month follow-up was completed for 16 patients (67 %).

At 6-month follow-up, five patients had died (31 %). Three patients died of a non-cardiovascular cause. Of these, one patient died due to sepsis with *S. epidermidis* without evidence of endocarditis at day 46, 1 due to renal failure at day 57, and one patient due to a community-acquired pneumonia at day 67. Of the cardiovascular causes, one patient died at home due to an unknown cause at day 99, and one patient due to decompensated right-sided cardiac failure with impaired liver function at day 57.

## Discussion

The available TAVI prostheses evolve rapidly, and various self-expanding, balloon-inflatable, and self-inflatable devices exist. In this paper, we describe our experiences with the JenaValve™ (JenaValve Technology GmbH, Munich), which received its CE approval for aortic stenosis in September 2011 and for aortic regurgitation in September 2013.

The JenaValve™ is a second-generation self-expandable device composed of a trileaflet porcine root valve mounted on nitinol double bowed struts with three positioning feelers. It is available in three sizes (23, 25, and 27 mm) in Europe, covering an aortic annulus range of 21–27 mm. This prosthesis can currently only be implanted by an anterograde approach, although a clinical trial for transfemoral delivery starts this year. The three positioning feelers give the JenaValve™ its capability to guide an exact positioning with the possibility of repositioning. Its method of anchoring, namely clamping itself around the native cusps, is very different from the radial force onto the aortic annulus and/or descending aorta used in for example the SAPIEN XT™ and CoreValve™ prostheses (Table 5).

**Table 5:** Differences in prosthesis characteristics

	CoreValve™	SAPIEN XT™	JenaValve™
Valve tissue	Porcine pericardium	Bovine pericardium	Porcine root
Stent	Nitinol frame	Cobalt chromium alloy	Nitinol frame
Valve location	Supra-annular	Intra-annular	Intra-annular
Implantation	Self expanding	Balloon expandable	Self-expanding
Route	Retrograde	Antegrade and retrograde	Antegrade
Delivery system	18 French	16 and 18 French	32 French

Differences between the CoreValve™, SAPIEN XT™, and JenaValve™ prosthesis

The procedure success in our cohort was 88 % (*N* = 21). This is comparable with the procedure success in the JenaValve™ CE-mark approval study: 82 % (seven conversions of procedure, five deaths). However, we experienced no cases of device malpositioning, conversion to surgery, conversion valve-in-valve, or conversion to another TAVI device. The positioning of the device could be performed in a very well-controlled manner and the ‘Cathlete plus’ delivery system allowed a step-by-step and controllable opening of the device.

The ‘real-world’ 30-day mortality rate after TAVI is approximately 7.5 to 8 %. [6–8] In transapical procedures, the mortality rate is generally higher compared with transarterial procedures (approximately 10–11 vs. 5–6 %) [7, 8]. These differences in mortality rate could partially be explained by the fact that transapical TAVI is usually performed on higher risk patients and that comorbidities interact differently per approach. Also, experience with the approach is related to mortality [9], and outcome improves with experience [10]. The CE-mark approval study for the JenaValve™ found a mortality rate of 7.6 % at 30 days (7 of 66 patients) [11]. In our cohort, 30-day mortality was 4 % (*N* = 1). This seems favourably low, although it could be partially explained by the small sample size. However, also the rate of the VARC defined safety endpoint (i.e. event-free survival) at 30 days was high (88 %, *N* = 21). This is comparable with the rate of safety endpoint after transfemoral TAVI [12]. The rate of PVL in our cohort also seemed promising, with only one case of moderate PVL (4 %). In the CE-mark approval study, PVL was seen in 53 % of patients (all mild or moderate). The low severity of PVL is encouraging, since not only moderate to severe but even mild PVL may impact mortality [13, 14].

One procedural disabling stroke occurred in a patient with severe extensive aortic calcification on dual antiplatelet therapy. Despite the availability of a protocol for antithrombotic therapy at our centre, the treatment regimen differed on discharge: not all patients received antiplatelet therapy [15]. This strategy is used by more centres, and seemed to have no negative implication in this population.

Conduction disturbances after TAVI are frequent and highly device dependent, with a five times higher incidence of new permanent pacemaker implantation after CoreValve™ compared with SAPIEN XT™ [7]. The rate of pacemaker implantation after TAVI was 13 % (*N* = 3) in our cohort, comparable to the CE-mark approval trial [11]. The observed lower rate compared with the CoreValve™ might be explained by the lower stent profile and its different method of anchoring, resulting in a reduced implantation depth in the left ventricular outflow tract and reduced radial forces at the level of the annulus and its surrounding tissue. However, the occurrence of new LBBB was seen in 38 % of cases in this series. This is of importance, since LBBB has been shown to be an independent and important risk factor for all-cause mortality after TAVI at 16 months [16]. No total incidence of new LBBB was documented in the CE-mark trial for the JenaValve™ [11] and future studies should address the rate and consequences of LBBB after implantation of the JenaValve™.

In our first experience, we find JenaValve™ an adequate prosthesis with a high success in implantation, also in less severely calcified aortic annuli. Since the JenaValve™ in fact grabs the native leaflets, this prosthesis might be indicated in non-calcified native valves such as in severe aortic regurgitation [17]. This is in contrast to the SAPIEN XT™ valve, which is anchored purely by radial force and needs some kind of calcification to plant itself and reduce the risk of valve embolisation and aortic annular rupture (Table 6). It seems that with novel devices like the JenaValve™, a more tailored approach to device selection with regard to anatomical factors could be applied.

**Table 6:** Suggested indications for SAPIEN XT and JenaValve in transapical approach

	SAPIEN XT™	JenaValve™
Aortic annulus range	18–27	21–27
Straight ascending aorta		≥65 mm
Distance between annulus and coronary ostia	≥10 mm	≥8 mm
Bicuspid aortic valve	++	--
Degenerated prosthesis	++	--
Porcelain aorta	++	-
Severe calcified nodules on native leaflet	+	+/−
Non-calcified aortic valve (e.g. AR)	-	+++
Small distance annulus to coronary ostia	+/−	++

With the development of novel TAVI devices, a more tailored approach to device selection with regard to anatomical factors could be applied

## Conclusion

In conclusion, transapical transcatheter implantation using the second-generation JenaValve™ prosthesis seemed adequate and safe in this first experience cohort. An advantage of this device is its precise anatomical positioning. Overall, the second generation JenaValve™ device offers a novel treatment option for TAVI.